# STLV-1-infected Japanese macaque as a model of HTLV-1 infection

**DOI:** 10.1186/1742-4690-11-S1-O12

**Published:** 2014-01-07

**Authors:** Michi Miura, Junko Tanabe, Kenji Sugata, Tiejun Zhao, Guangyong Ma, Paola Miyazato, Jun-ichiro Yasunaga, Masao Matsuoka

**Affiliations:** 1 Institute for Virus Research, Kyoto University, Kyoto, Japan

## 

Various non-human primates are the natural hosts of simian T-cell leukemia virus type 1 (STLV-1). In the present study, we analyzed Japanese macaques naturally infected with STLV-1, and evaluated them as an animal model for HTLV-1 research. Approximately 60% of individuals in the colony are seropositive for STLV-1. Clonal proliferation of STLV-1^+^ cells was investigated by massively sequencing the provirus integration sites. We found that some clones proliferated distinctively in monkeys with higher proviral load. T lymphocytes expressing Tax in the peripheral blood were largely CD4+. Notably, one of the monkeys surveyed in this study developed T-cell lymphoma in the brain, indicating that STLV-1 is oncogenic in Japanese macaques. We also assessed the molecular function of STLV-1 Tax and STLV-1 bZIP factor (SBZ). STLV-1 Tax activated NFAT, AP-1, canonical Wnt and canonical NF-kappa B pathways, whereas SBZ suppressed those signaling pathways. SBZ enhanced TGF-beta signaling, but STLV-1 Tax suppressed it. These findings suggest that STLV-1 Tax and SBZ have similar functions to their counterparts of HTLV-1. In addition, we found that administration of anti-CCR4 antibody, which is currently used in Japan for the treatment of ATL patients, efficiently reduced proviral load in STLV-1-infected Japanese macaques. Our study provides the evidence that Japanese macaques naturally infected with STLV-1 correspond to HTLV-1 carriers and are a suitable animal model to investigate the pathogenesis of HTLV-1 and novel therapeutic strategies.

